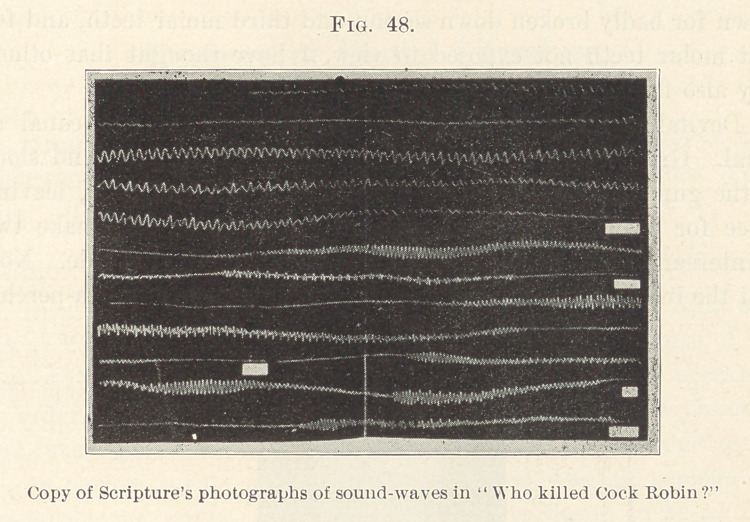# A System for the Surgical Correction of Harelip and Cleft Palate

**Published:** 1905-07

**Authors:** Geo. V. I. Brown

**Affiliations:** Milwaukee, Wis.


### International Dental Journal.

VOL. XXVI. JULY, 1905. No. 7.

### Original Communications.[1]

### A SYSTEM FOR THE SURGICAL CORRECTION OF HARELIP AND CLEFT PALATE.[2]

BY GEO. V. I. BROWN, A.B., D.D.S., M.D., C.M., MILWAUKEE, WIS.

THE reasons for the existence of any system of surgical treatment, and particularly one applied to the correction of a special class of affections, must be, first, a demand for such service; second, its necessity by reason of the large number of failures and extreme difficulty of accomplishing good results by other methods; third, that it makes possible an enlargement of the field of surgical accomplishment in at least one direction.

The blind, the deaf and dumb, the tuberculous, and the crippled almost everywhere have institutions established for their care. States and communities have recognized the duty of ministering to the comforts and necessities of the afflicted in these

[1] The editor and publishers are not responsible for the views of authors of papers published in this department, nor for any claim to novelty, or otherwise, that may be made by them. No papers will be received for this department that have appeared in any other journal published in the country.

[2] Read in the Section on Stomatology of the American Medical Association, at the fifty-fifth annual session, June, 1904. The article has been abbreviated by the omission of part of the illustrations. The entire article appears in the reprints. Copied, by consent, from the Journal of the American Medical Association.

directions. Large sums are annually contributed for the maintenance of such work and the encouragement of scientific investigation to increase the efficiency of its usefulness, but no such responsibility has been assumed by municipalities, nor has any notable portion of endowments of charitably inclined wealthy citizens been devoted to the relief of these unfortunate individuals, half dumb through malformation of their mouths and palates and doubly cursed by having the deformity and the even greater affliction of mental comprehension of difference from their fellows. Their harelips, scarred and ill-shaped faces, mark them, through no fault of their own, as were branded the felons in barbaric times; speech communion with their fellows is almost wholly forbidden, and they live ever in the dark shadow of misfortune.

NECESSITY FOR NEW AND SYSTEMATIC METHODS.

To those familiar with the literature and practical features of this subject it is patent that all methods of treatment, whether prosthetic or surgical, have hitherto failed to be successful in a large number of cases, and have not been able to meet the demands of many cases at all.

Numerous methods of operation and most ingenious forms of obturators have been from time to time suggested by authors, and adopted by practitioners, with more or less benefit in individual or special kinds of cases, and yet a systematic classification of the forms, with particular methods to be employed in the correction of each variety, showing comprehensive consideration of the distinct requirements that are found to be imperative, seems not to have received the attention that is due. This the system that I now present to you in my own name, after years of practical study in the care of a large number of patients, seeks to accomplish in a considerable measure.

Its methods are necessarily founded on the ground-work constructed by eminent surgeons who have contributed to literature the results of their efforts in this direction, beginning probably with Lemonier, in 1776, as the first to attempt to close palate fissures by surgical means, followed by Eustache in 1799, von Graefe in 1876, Roux in 1879, Warren, of Boston, in 1820. Diffenbach, Liston, Sir William Fergusson,[1] the real author of successful staphylor-

[1] International Clinics, 1877.

raphy; Sedilot, Pancoast, William, Garretson, Wolf,[1] Erdman, Billroth,[2] von Langenbeck, Trilet (1889), and a host of great sur-

Fig. 1.

Cast of the mouth of a baby with single harelip, as in Fig. 2, with the characteristic enlarged turbinate.

Fig. 2.

Baby with single harelip. Nose flattened and drawn to one side.

geons, past and present, who, despite great discouragement,—for this work is ever discouraging to surgical procedures when com-

[1] Archives of Klinical.
[2] Vienna Medical Presse, 1889.

pared with other fields of operation,—have each lent some share of personal skill and ingenuity to aid the perfection of the operative technic necessary to insure the ultimate success of the work.

### ORIGINALITY.

The operation for reduction of the width of the fissure, in combination with the use of the appliance as described, is original with me, and I first performed it in 1897.

The methods of preparatory treatment of infants for the correction of facial deformity in lip cases are, so far as may be judged from careful study of the literature, also original with me.

Various minor steps in the operative technic have been developed from time to time, without previous knowledge of others having introduced them, and are believed to be original.

### CLASSIFICATION.

The following is a classification that I have previously reported in other papers, and is designed to cover the important considerations from a surgical point of view:[1]

1. Character: Congenital acquired.
2. Form:

   Fissure in velum palati only (Figs. 32 and 34).

   Fissure of the velum, including part of the hard palate also (Fig. 36).

   Cleft entirely through both hard and soft palates (Fig. 39).

   Median fissure through both hard and soft palates, with bifurcation at the maxillary bone (Fig. 43).

   Double separation divided by the vomer, including the hard palate, with wide fissure almost completely obliterating the velum (Fig. 14).
3. Age:

   At birth or during early infancy (Fig. 1).

   Six months to one year old (Fig. 4).

   Two years old and during the period after deciduous teeth have been erupted, but before their permanent successors have caused them to loosen (Figs. 36 and 39).

---

[1] Brown, George V. I., "Surgical Correction of Malformations and Speech Defects due to or associated with Harelip and Cleft Palate," Jour. A. M. A., 1902. "A System for the Treatment of Harelip and Cleft Palate," Chicago Med. Record, 1903. "Relation and Comparative Merits of Surgery and Prosthesis in the Correction of Harelip and Cleft Palate," Dental Summary, 1903.

Twelve to eighteen years of age, or later years. After permanent teeth have been erupted, but before developmental processes have been completed.

Adults with teeth in upper jaws (Fig. 43).

Adults with edentulous upper jaws (Fig. 18).

Fig. 3.

Photograph of same baby shown in Fig 2 after operation, and intended to call attention to the straightness of the nose, equality of shape of alæ, and the fact that the mouth is closed, which indicates that he breathes naturally through the nose.

Either lip or palate may be imperfect without the other being affected, although, as will be noted by study of the illustration, it is common for certain forms of lip and palate deformities to be associated.

Congenital lip deformities are either unilateral (Fig. 2) or bilateral (Figs. 7 and 11). The former may be of first degree, or little more than a notch; of second degree, with a fissure extending completely through both lip and maxillary bones; or of third degree, a wider separation with unilateral protrusion of the intermaxillary bone.

Bilateral or double harelip has usually marked deformity of the vomer with protrusion. To these, unfortunately, must be added the cases that have been imperfectly operated on in early life, which so often call for correction and which, although presenting an infinite variety of imperfections, have nevertheless certain characteristic defects that admit of distinct recognition and may be enumerated as follows: 1. Notch at the labial border. 2. Deflection of the tip of the nose, with deviation of the cartilaginous septum and flatness of the ala on the affected side. 3. Unsightly scars. 4.

FIG. 4.

Six months to one year old.

Stenosis of one or both nares. 5. Marked arrest of development due to removal of the intermaxillary portion of jaw.

EARLY OPERATION.

Radical operation for closure of cleft palate in the infant is to be condemned, because the death-rate is unquestionably high. This is true of every serious operation at a period of life when the vital forces are busy adjusting themselves to a new, though natural, environment. The marked change from the direct protection and blood-supply of the uterus to the uncertainties of nourishment and temperature after birth involves a problem fraught with many difficulties of solution, even in the care of normal children. How much greater, then, when the vital forces are sapped by hemorrhage, and the digestive function disturbed by bacterial influences incident to operative procedures in the oral cavity, or wires left in the tissues

FIG. 7.

Baby with double harelip and the usual oral, facial, and nasal distortions.

FIG. 8.

Same baby as in Fig. 7, with parts bound in position after preparatory operation on the vomer and manipulation to replace parts and to bring them into normal position before final operation.

of the mouth and jaws to gather bacteria in spite of antiseptic precautions, as they must and always do under these conditions.

Fig. 9.

Same baby as in Figs. 7 and 8 and after preparatory operation and treatment, with parts ready for closure by final operation.

Fig. 10.

Another view of same baby pictured in Figs. 7 to 9, after treatment, showing that the sides of the fissures remain in contact without sutures, and calling attention to the improvement of the nose. Child is now ready for final operation on lip.

The question of meningitis also becomes a serious consideration when one contemplates the result of crushing the maxillary bones together to approximate the sides of such wide fissures as are found to exist in many cases, because of the correlation of the still unformed semi-cartilaginous bones of the face and head, which share in the effect of compression, and added to this is the danger of infection being carried to the brain through the direct channels of communication.

Fig. 11.

Baby five weeks old, with characteristic deformity of double harelip.

Finally, it may be said that the results of such operations from a cosmetic point of view are certainly not so perfect or pleasing as may be accomplished by other means, and it is, moreover, reasonably certain that the functional possibilities of the palates and mouths thus treated with regard to speech are often not of the best.

Foregoing statements may be better understood and their importance appreciated more fully, also the reasons for methods of treatment described later by a study of the defective operative results illustrated in Figs. 20, 24, 25, 26, 29, and 30, which have been taken from cases in practice, and which may, therefore, be said to be essentially practical.

The excessive unilateral development is a marked and almost invariable condition in these cases, the more so because arrested development of the opposite side serves to increase this deformity.

The enlarged turbinated, or the appearance of enlargement, which may be noted in each of the pictures and casts, is a very common concomitant, and doubtless is due to a partial unfolding of its curative, in an attempted adjustment to the unusual nasal space occasioned by the fissures.

If, therefore, the sides of the jaws of any such cases are crushed together by immediate closure, as is recommended by some authors, one side of the face will project beyond the other, giving a peaked,

Fig. 13.

Front view, after operation, of baby shown in Figs. 11 and 12.

extremely unpleasant appearance, that later developments can never hope wholly to overcome.

It must be apparent also that in many instances it is the inferior turbinated bone that must meet in direct line the border of the maxillary portion of the palate on the opposite side, or if not this, then there will be a complete and irreparable permanent stenosis of the naris, on the affected side, at least.

Fig. 26 shows a little boy whose palate was closed in early infancy, and, as may be seen, the breathing is entirely by mouth. There is complete obliteration of one nasal passage, and almost the same condition of the other. The appearance of the face also confirms my statements.

Fig. 14.

Double separation divided by the vomer.

Fig. 18.

Cast of mouth of young lady shown in Figs. 20, 21, 22, and 23, with cleft extending through both hard and soft palates, the fatal error of extracting all teeth having been made some years before coming to me. Line is drawn where maxillary bone was sawed and fractured to correct unequal maxillary development, with result as shown in Fig. 23.

In these cases the intermaxillary bone is either attached to one side or separated by fissures on both sides. In the former case it projects, as shown in Fig. 2; in the latter, as illustrated by Figs. 7, 11, and 15. Thus an immediate, direct and forcible closing of the fissures would, under both conditions, leave an unsightly protrusion of one side, or the anterior aspect of the mouth and nose, which would be all the more noticeable because of the enforced narrowness.

FIG. 20.

Patient twenty-two years old, the cast of whose mouth is shown in Fig. 18. Although the lip was closed in infancy by a famous surgeon whose skill is beyond criticism, the failure to adjust the maxillary bones resulted, as may be noted, in great nasal and facial deformity. Ala flat and nose almost entirely on one side of the face.

One would expect speech to be facilitated by the fact that when early operation has been performed no opportunity is given to acquire the characteristic defects common to individuals afflicted with imperfect palates, and yet we usually find that this is not the case, owing to insufficient room for the tongue and conditions due to hasty closure of the soft palate, which must of necessity be less perfect than at a later age when more time can safely be given to accurate adjustment of the parts.

The impossibility of preventing infants from crying and straining the muscles of the soft palate, thus endangering the pulling out of stitches, together with the danger to the life of the child, both tend to show that there is real need for other methods.

TREATMENT OF INFANTS.

Provision has been made for all, or, at least, most of the contingencies that arise in the care of these infants. If a child can be

Fig. 21.

Same view of patient shown in Fig. 20 after lip had been reoperated on, scar tissue removed, and both jaw and nose straightened.

placed under treatment immediately or shortly after birth, when both lip and palate are affected, the method of procedure is as follows:

A day or two is given to allow the infant to become accustomed to its surroundings, if in a hospital, as is best, although not absolutely necessary. The child is weighed and its general condition of health observed, character of fecal passages, etc. If conditions are favorable, strips are then adjusted to prevent the action of the lower jaw from forcing the maxillary bones apart and widening both the palatal fissures and the lip separation. This also brings into play the muscles in such manner as to produce a narrowing effect whenever the little patient laughs or cries; in other words, the forces that ordinarily increase the deformity are now engaged in its reduction, and, in addition thereto, are stretching the lip muscles and skin surfaces on each side, so that in a very short time there will be an abundance of material to bridge across and make a sightly operation, where formerly the supply was perhaps so scant as to make an operation extremely hazardous.

FIG. 22.

Side view of Fig. 20, to be compared with Fig. 23.

It is necessary that it be trained to eat from a spoon, or dropper, and all habit of sucking should be overcome, if possible, before operation.

Figs. 2 to 16, inclusive, call attention to the practical results of such treatment, and I think all will agree that a few weeks' preparatory care may sometimes be beneficially employed.

AGE FOR PALATE CLOSURE.

The proper time for palate operation depends largely on the physical development and condition of health of the individual, also on training, and subjection to proper control, because the best of health is not only desirable, but imperative, and unless the assistance of the patient can be secured in the way of intelligent co-operation in the post-operative care and treatment, it is most unlikely that good results will be accomplished. Therefore we find

Fig. 23.

Side view of Fig. 20, showing ala of nose on right side to be the same shape as on the left, as shown in Fig. 22.

that some children can be operated on safely and satisfactorily at a much earlier age than others. Yet it is by all means desirable that no bad speech habits be given time to establish themselves.

Our choice must then come between the years when the deciduous teeth have been fully erupted and before they have become loosened for their permanent successors, and yet, before speech habit has been fully formed. Fortunately the period during which

Fig. 24.

Double harelip.

most children talk "baby talk," as it is called, gives us more or less opportunity to make a satisfactory decision, since during this time the manner and form of speech are not what they will be later, and one change or another is much the same, because correct speech is yet to be acquired in any event.

REDUCTION OF WIDTH OF FISSURE BY THE USE OF AN APPLIANCE.

Having thus determined on the question of time, we next attach an appliance, such as is shown in Fig. 44. No other agent is necessary at this age, as the bones are soft and yielding, so that a slight turn of the little screw two or three times daily is sufficient to materially reduce the width of the fissure and to give the increased angles of the palate surface, which are necessary to make operation simple.

PREPARATORY TREATMENT OF ORAL, NASAL, AND FAUCIAL TISSUES.

During the time of wearing the appliance, daily irrigation with nasal douches of salt water, boric acid, or other mild solutions of like character should be given to correct the diseased condition of the nasal mucous membrane.

This treatment is important in all cases, for whether the patient be young or adult, hypertrophic rhinitis is a natural result of unusual exposure of the nasal and pharyngeal mucous membrane to thermal changes, irritation from mouth bacteria and other irritants.

Fig. 25.

Shows deformed facial appearance, under conditions represented in Fig. 24, after intermaxillary bone has been removed to close double harelip.

Manipulation also should be given when tendency to gag on irritation of the faucial surfaces exists; otherwise, retching or vomiting at the slightest touch will make it almost impracticable to thoroughly cleanse the wound surface of the palate down to the tip of the uvula, as should be done without exciting such disturbances, which might cause destruction of the integrity of the stitches. Proper treatment is of inestimable benefit in these two directions.

### TREATMENT AT SIX MONTHS OR OLDER.

Infants who have reached the age of six months or more sometimes require the assistance of wire passed through the lateral sections of the divided jaw and secured by lead or silver plates, in order that by a slight bending of these wires from day to day a sufficient tension may be produced to bring about a readjustment of the deformed structures.

YOUNG AND ADULT CASES.

The appliance used for attachment to the teeth, and the nut, with threaded metal bar, do not differ materially in young or

FIG. 26.

adult cases, except that a trifle more strength may often be required for the latter. There is, however, a difference in the manner of application. The yielding quality of the young bones makes it possible, by steadily exerted pressure of the appliance alone, to bring the sides nearer, and thus to reduce the width of the fissure; at the same time the palatal arch is increased in height and the sides so shaped as to make the angle of their slant more acute; for older patients this is best accomplished by cutting along the external walls of the superior maxillary bones, through the outer plates, particularly at the malar process, and behind the tuberosities. With strong forceps then crush together until a partial fracture

Fig. 27.

Front view of same boy shown in Figs. 25 and 26, after operation.

makes exact adjustment to proper form an immediate possibility. The appliance, as in Fig. 45, then acts as a splint capable of exerting pressure if needed.

It is most desirable that the anterior or narrower portions of the fissure should be brought into direct contact, sufficiently so, if possible, to allow of freshening not only the soft tissue, but the bony borders as well.

The complete circulation thus secured, after union has taken place, serves to nourish the flaps when the muco-periosteal final operation is performed, and does much to prevent sloughing, which is so much to be dreaded.

IMPROVED FORMS OF NEEDLES.

The curve of each needle should be as exactly fitted to the part of the operation for which it is to be used as possible, be-

FIG. 28.

Side view of boy shown in Figs. 25, 26, and 27 after operation and restoration to more natural lines in profile.

cause, in describing the segment of a circle necessary to insert a suture in the anterior portion of the hard palate, where it is high and narrow, and in the posterior portion of the soft palate, where the space is broad and shallow, the requirements are entirely different. This is true also of the intermediate portions of the palate surface. The mucous secretion and hemorrhage make it difficult to see perfectly at all times in the act of catching the point of the needle and to easily secure the suture. Therefore great assistance is rendered if the exact curve of the needle can be so calculated as to enable the operator to know, with some degree of accuracy, just where the point will reappear on the side opposite the point of insertion.

Three sizes of needles, after the form of perineum needles, curved to allow of convenient use at the different angles required for operations in the mouth, meet the necessities of the situation very well, but for my own use it is a great advantage, and since I use both right and left hands in operating, much benefit is derived and the time of operation materially shortened by the use of

Fig. 29.

Young man of twenty-two, showing result of early imperfect operation for double harelip.

a set of five pairs of needles (my own design), with opposite curves (right and left), each pair graded in size from the large one used in the posterior portion of the soft palate to the smallest, which are suitable for the extreme anterior portion of the hard palate. These are threaded with the suture materials before operation, in order to save time and confusion, and to leave the assistant quite free to handle sponges and thus reduce to a minimum the troublesome hemorrhage that usually attends operations in this region.

SPEECH RESULTS AND TRAINING.

A system would be but half complete that offered no assistance beyond repair of the imperfect oral instrument and did nothing afterwards to teach the use of it.

Post-operative training must be our final consideration. It is a noticeable fact that individuals who are quite unable to recognize imperfections in the sounds of words, as they pronounce them, detect the difference at once when their own speech records are sounded from a graphophone, the effect being the same as though another person were speaking. To take advantage of this it is advisable to have a record taken before and another immediately after operation, while from time to time, as the training progresses, much encouragement is given by having others taken for comparison. Preferably, the same words should be repeated in order that the test may be exact.

Walter Edward Scripture, in his most valuable work, "The Elements of Experimental Phonetics," makes the following reference to phonographic speech records, which is completely in accord with my practical experience in training these cases:

> "When a record is found that speaks clearly, in a natural voice, it can be trusted for what it says, since it cannot say anything more than is on it, and cannot improve its own tracing. The speech represented by a tracing from a record is the speech of the record itself. How nearly this reproduces the original speech can only be determined by comparing it by the ear with the words of the original speaker. By skilful manipulation records can be made whose speech cannot be distinguished from that of a living person, except by their weakness and by the scratching noise due to the friction of the tracing point in the groove."

A study of phonographic records, taken from patients of different ages, temperament, degrees of intelligence, and extent of palate deformity, showed some interesting differences in speech defect.

The facility with which some patients improve in speech after restoration of the palate by operation, and the difficulties that others of the same age and equal mental capacity seem to encounter, as noted by comparison of the phonographic records and casts, indicates very clearly that the extent of the fissure is by no means an exact index to the character of speech defect, there being a somewhat surprising dissimilarity of speech sounds among these patients. Undoubtedly there are certain easily recognizable wrong sounds common to them all, but the inability to speak correctly individual letters, words, and sentences is by no means the same in all cases, even where the palate fissures are quite similar; all of which serves to emphasize the necessity for careful consideration of speech mechanism as absolutely a first essential in the construction of a plan for speech and voice training calculated to give the best results.

Fig. 30.

Front view of same case as in Fig. 29.

This, on previous occasions, I have summed up in the following manner:

The first cry of the child is merely a sound caused by reflex action of the muscles, without any guiding influence exerted by the faculty of reason. This is followed by the first efforts of sound to represent intelligent words as objects begin to be recognized, and gradually this is continued and extended until expression of ideas in speech becomes possible. It will be readily understood that if these efforts have been on normal or correct lines, the muscular activity necessary to sound-producing must have been guided by the proper nerve-centres, which will have caused an increase in the brain development of those centres, and the messages sent from the motor tract to the muscles which are concerned

Fig. 31.

Result in case shown in Figs. 29 and 30 after operation, by which lip, nose, and jaw were readjusted and scar tissue removed.

in the utterance of words will be in all respects correct, and the habit of proper speech will have become an established fact. On the other hand, however, if a deformity has existed from birth, by reason of which the normal use of certain muscles has been greatly restricted, and the use of certain other muscles, not commonly used in the process of word enunciation, has received more stimulation than would have been the case had there been a perfectly formed mouth and throat, the results must invariably be an increased development of the nerve-centres which are injurious to speech, with a faulty development of those that are necessary to perfect speech. This might be termed a habit, but the word habit conveys too restricted an idea of the condition. For example, when the eye, through its retinal image, registers on the brain structures the particular nerve stimulations which in time will become associated with the name of an object, its form record is established by

FIG. 32.

Fissure in velum palati only.

what may be known as the visual memory centres. Precisely also are the somæsthetic areas affected by the tactile sense, and memory of the sense of touch, as well as taste, smell, and other stimuli that may have been excited by or associated with any particular object, and, when the sensorium takes consciousness of this object, the name of which has become known to it, there is required the co-ordination between these different memory centres in order that the proper message may be sent to the motor centres, through which certain muscles may be set in motion in the proper manner to produce the sound which may be clearly recognized as the spoken name of the object.

It is known that in speech the muscles of the chest, which are responsible for expiration, the muscles that raise and lower the larnyx, those that tighten the vocal cords and tip the hyoid bone, as well as resounding properties due to the nearness of the spinal column, and the co-operation of the forces that are applied in raising and lowering the soft palate, the adjustment of the tongue and proper action of the muscles of the cheeks and lips, are all

Fig. 36.

Cast of mouth of child two and one-half years old, with cleft including the velum and parts of the hard palate.

necessary for the utterance of even a single word. If, therefore, during the life of the individual, through faulty operation or adverse action of these agencies, wrong messages have been constantly sent to portions of the brain concerned in making a certain sound, and if the auditory memory centres have registered, by the constant hearing, imperfect sounds for specific words, which accordingly have caused the development of brain structure which is all active against correct speech, and if there be an insufficient development of those centres which are needed for perfect speech, how great becomes the difficulty of giving a speech power to individuals in the face of all these acquired disadvantages.

Kingsley's palatograms (Fig. 47), obtained by coating the surfaces of thin plates covering the palate surfaces with chalk, in such manner that whenever the tongue touched, in sounding a word or letter, the black surface showed through, are of interest as indicating why certain letters are so troublesome in these cases. It will be noticed by the illustration that points of contact of K and G are post velar, and therefore not only practically impossible without a velum, but almost equally so, unless after operation the soft palate secured be of perfect form and sufficiently flexible, under muscular control, to allow this contact to take place. These, it will be remembered, in support of the accuracy of Kingsley's work, are invariably troublesome sounds. Foster and Reichert, in describing speech under the heads of vowels and consonants, divided into explosives and aspirants, vibratory and resonants, giving in

Fig. 37.

Cast of same patient's mouth as in Fig. 36, after closure by operation.

detail the mechanism of alphabetical sounds, conclude with the following statement, that, from our point of view, is all important:

> "On many of the above points, however, there are great differences of opinion, the discussion of which, as well as of other more rare consonantal sounds, would lead us too far away from the purpose of this book. The following tabular statement must therefore be regarded as introduced for convenience only."

Scripture calls attention to the following factors of voice control:

"1. Reflex tonus: Tonus is a faint muscular contraction due to continuous weak nerve stimulations, easily subject to fatigue, ill health, and other demoralizing conditions, lack of or disarrangement of which causes marked change of voice in both speaking and singing.

"2. Force of movement: This depends on the amount of stimulus sent to muscles, movements of which include not only those directly involved, but also their antagonists. This requires an excess of effort over what might be expected, but when the innervations are properly co-ordinated this excess is not necessarily large and fatiguing.

"3. Accuracy of movement: Inaccuracy of movement is a fundamental source of inaccurate and wrong sounds.

FIG. 39.

Cast of mouth of boy two and one-half years old. Cleft through hard and soft palates.

"4. Precision of movement: This refers to regularity and evenness of execution and depends on nervous control.

"5. Accuracy and precision of co-ordination: This represents the nervous control over simultaneous muscular movements. Some forms of thick speech of alcoholic intoxication and incorrect adjustments during excitement are caused by defective co-ordination in speech effort.

"6. Quickness of response: This is action of the nervous centres that tends to become automatic. One object in vocal training should be to render speech and song automatic.

" 7. Quickness of muscular movement: This depends on both muscular and nervous quickness and must be properly balanced; otherwise, speech appears labored or slurred.

" 8. Auditory motor control: The learning of speech sounds consists largely in forming connections between motor and auditory sensations.

Fig. 40.

Cast of same mouth as Fig. 39, showing result after operation. Fissure reduced by the use of an appliance and closed by final operation.

" 9. Ideomotor control: Sounds occurring simultaneously with sights, touches, tastes, smells, emotion, act of will, etc., tend to be connected with them, so that when any one of a complex group occurs again, the others are revived more or less clearly in consciousness. It is in this way that speech movements become associated with printed letters.

" 10. General voluntary control: This is subject to changes of nutrition, fatigue, emotion, and general habits, on all of which vocal control must place its dependence."

In my practical training of these cases, I have found that one of the best aids to speech improvement is singing. Even though the sounds be less musical than might be wished, and they often are, vocal lessons, or, in the absence of this, constant use of the voice in chanting sentences, after the manner of intoning church services, will enable the individual to speak reasonably well the particular sentences practised in this manner in a very short time. Combining, as these exercises do, most of the essential points brought out in the foregoing description of speech requirements, it follows that there can be no more beneficial procedure.

Nervousness and over anxiety about rapid improvement in many cases retard progress quite seriously, and must be overcome in so far as possible at the very outset. Such patients are best counselled that improvement must necessarily be slow, and cautioned against making too great effort.

A large majority of these patients are, as might be expected, individuals of extremely unstable nervous organization, and it is of quite frequent occurrence that some of the neuroses common to neurotics occur in the way of speech impediment. Aphasia with regard to certain letters and words, stammering, nervous hurrying, together with letters and sounds, are very often complications that retard progress. Doubtless much confusion in reporting results has occurred through failure to distinguish these affections.

Exactly why interference on the part of any nervous agent that might tend to disturb the rhythm of muscular movement must necessarily disarrange the speech harmony is most beautifully shown by a copy of one of Scripture's plates, which is a reproduction of a photograph of actual sound waves taken during the recital of "Who killed Cock Robin?" (Fig. 48).

### PHONOGRAPHIC RECORDS.

The phonographic records show the degree of improvement in speech.

Case I.—In the case of a girl of fourteen, who had only a fissure in the soft palate, before operation, it was impossible to distinguish speech sounds sufficiently for unaccustomed persons to understand what she said. Therefore, a phonographic record would have been a mere jargon of unintelligible sounds. The fourth grade was as high as she had been able to get in the public schools, because teachers could not understand her. Immediately after operation marked improvement was noted by record. Later records showed remarkable change for the better, and on one or two occasions, after training, she has recited before large audiences, with much clearness and a very good approximation of perfection of sound, verses on which she had been drilled, but in general conversation her progress was not in proportion to that of better educated patients, as was noted in records of other cases. Eagerness in conversation always tended to cause lapses into old speech habits, and in this case, such difficulty was hard to overcome; a difference being apparent if she were allowed to associate for a time with people who themselves spoke incorrectly or carelessly or the reverse.

FIG. 43.

Photograph of mouth of patient with cleft through both hard and soft palates. Casts shown in Figs. 41 and 42 were taken from this patient's mouth.

CASE II.—Another young girl, twenty-two years old, with the same character of fissure, but who had a high school education, could speak much better than the preceding one before operation, the range of her intelligible, even though somewhat nasal, sounds being much less limited. After closure of the cleft, though operative results were quite perfect in both cases, her progress in the line of improvement in conversational sounds was shown by the record to be much more marked.

CASE III.—Patient, mother of a grown-up family, aged thirty-eight. Cleft in soft palate the same as Cases I. and II. She was not well educated, but not ignorant, and of less nervous temperament than the other two. Speech, as might be expected, was better than Case I. and not so good as Case II. Soon after operation, therefore, without training, her record was found to be surprisingly good, due in a considerable measure, no doubt, to freedom from nervousness. Later reports of improvement were much more favorable than was expected, but have not yet been confirmed by record.

Case IV.—This patient, a girl of sixteen, in whom the fissure was confined to the velum, as in all the preceding cases, was as uneducated as the first patient, but with less natural intelligence. Scarcely a single word in her first record could be understood. Later, and after much training, she was able to recite simple rhymes before large audiences quite well, but she has never acquired good speech. When corrected, she can repeat, after another, sentence by sentence, even most difficult words with little noticeable speech defect.

Case V.—A girl of twenty-two, who had an acquired fissure of the velum, due to hereditary syphilis, was operated on after preparatory administration of potassium iodide, with successful result, so far as closing the opening was concerned. It was not perfect in the sense that the preceding cases were, because cicatricial contractions, due to previous ulcerative processes, had stiffened the tissue. Notwithstanding the fact that this deformity was acquired at about the age of fourteen, and the patient's education was above the average, there was less improvement after operation than in any of the other cases.

Case VI.—In contradistinction to these other cases, a little boy of nine years of age, whose congenital cleft in the velum was like that in Case V., with very imperfect speech, was able to improve so rapidly that between the months of May and November defects were so overcome that his school-teacher did not notice unusual difference from other children of the same age.

Case VII.—A young woman of twenty-two, fairly well educated, with opening in velum palati alone, in whom speech sounds were very bad before operation, was able, by reason of good ear and singing practice, to improve sufficiently to be able to pray and sing alone at Salvation Army meetings within a few months from time of closure. In this case, undoubtedly, religious zeal helped to overcome self-consciousness, together with other mental and nervous hinderances, while constant attendance on the meetings of the army gave the best possible training to the vocal apparatus.

Case VIII.—A young man of eighteen had a complete cleft of both hard and soft palates. He is a graduate of a high school. Before operation it was almost impossible to understand him, yet, in repeating the alphabet, unusual ability to pronounce each letter was noted, even the G, K, and C being more than ordinarily good. Stammering was the prime cause of his speech difficulty. The record, taken two days after the last stitches were removed, complete union by first intention having taken place throughout, even to the tip of the uvula, showed an almost astonishing result, since his voice from the graphophone, singing the "Holy City," sounded better, perhaps, than many of our own would if a similar record had been taken.

Case IX.—A young man nineteen years old, with a fair education, had fissures through both hard and soft palates. The first speech record was better than Case VIII., on account of freedom from nervous habit, but ability to make separate sounds was less perfect. He could sing "Rock of Ages" quite well, and showed great speech improvement in later records as a result of two weeks' singing and drill exercise.

Case X.—A boy of nine, for whom only the preparatory operation has yet been performed, has given us two interesting records. In one he recites the Lord's prayer with an obturator in his mouth, and in the next equally well without it, yet, with cleft through both hard and soft palates, it must be understood he could not have learned to speak so well had he not had the mechanical assistance in the beginning.

CASE XI.—A young woman of twenty-two, highly educated, but with wide cleft through both hard and soft palates, made worse by having had several previous unsuccessful operations, improved so rapidly after the normal form of the palate had been restored by operation that she successfully passed an examination to teach in an Eastern law-school. Although records in this case are not complete, the fact that she now holds an important position and daily transacts the telephone business of a large establishment is sufficient proof of her improvement. Undoubtedly, her rare intelligence and persistence have been assisting factors.

FIG. 44.

**Cast with appliance adjusted as used in the reduction of the width of palate fissure, in cases illustrated in Figs 39 to 43, and all similar others of the same classes.**

CONCLUSIONS.

I have thus presented a number of cases, covering not only different forms of cleft palate deformity, but varying characteristics with the same classes of deformity, due to the several conditions that govern speech habit in these individuals. A large number of other cases of which I have from time to time taken record, under all the various forms and conditions of these patients, confirm the results given in those here cited. All the selections given in these illustrations have been made on the basis of demonstrating distinctive, important differences bearing on operative procedures, and speech results, rather than a multiplication of similar cases, that would involve endless repetition and confuse rather than make the subject clear.

It has been demonstrated that some of these patients, with openings so wide that there appeared to be almost no palate at all, spoke as well or better than others with only a slight velar defect, and individual distinctions have thus been made apparent, from all of which the following conclusions are obvious:

Fig. 46.

Photograph of patient with palate fissure, as in Fig. 43, after reduction by the use of appliance and final operation, with perfect result throughout the entire extent of both hard and soft palates down to the tip of the uvula.

To get the best results from operations, the palate must be restored in as nearly perfect form as possible. Freedom from scar tissue and from muscular tension sufficient to allow necessary speech movement are essential.

Proper muscular alignment at the point of union greatly facilitates the early usefulness of antagonizing muscles; therefore natural physiologic action is more promptly in evidence.

All these operative conditions are favored by the methods of the system herein described. Individual benefits, assuming that operative results have been perfect or as nearly so as possible, will be governed by patience, earnestness, and application of the patient. Natural intelligence, aided by education, will be manifest in assisting progress. A musical ear (so-called) and ability to note rhythm and time in music are of very great assistance.

Fig. 47.

Kingsley's palatograms.

No cleft palate deformity can be so bad that it cannot be corrected by surgical operation, nor need age prevent unless through lack of health on the part of the patient.

It has been my purpose throughout to treat this subject from the broad stand-point of a system to emphasize by precept and illustration the crying need for better treatment of a class of individuals whose misfortunes cannot fail to appeal to every one; to establish an understanding of the necessity for differentiation in the choice of operation and methods of treatment; and, above all, to prove the value of a practical system. Thus matters belonging strictly to surgical techniç have been, so far as possible, avoided and left for a future paper, but the post-operative treatment of these patients is so vital to the success of every operation that more than passing notice seems to be imperative.

It is too often taken for granted that more or less sloughing and pus formation must follow extensive mouth operations, and that surgical asepsis is impossible. In a sense, this must be admitted to be true, owing to natural anatomic obstacles to complete sterilization and the constant exposure to infection from so many sources, but, notwithstanding all this, most gratifying results can be secured, and so nearly a true primary union obtained as to make its essential benefit the same even with extensive wound surfaces. With the periosteum stripped from the palate surfaces; incisions reducing circulation to the farthest safe limit; nasal secretions above in contact with raw surface; mouth secretions below, mixed, as often occurs, with gastric regurgitations and vomited matter; only a comparatively thin veil of tissue bridging the space of the palatal separation of the bones and at the velum, exposed to destructive influences at every movement of the tongue or act of swallowing, it goes without saying that only the most rigid adherence to antiseptic surgical care could be effective.

Strong solutions of poisonous, or tissue destructive, germicidal agents are necessarily precluded in the mouth. Dilution in the oral fluids renders otherwise effective solutions of practically no benefit. The histologic character of the nasal, oral, and pharyngeal mucous membrane surfaces render sterilization extremely difficult, and it has been conclusively proved that animal fats, dead mucous cells, and other surface coatings resist even powerful drugs to such an extent as to protect underlying bacteria, while germs on the immediate surface are destroyed. Mechanical cleansing, therefore, is a first necessity, and next to this, frequent use of non-toxic or mild solutions of otherwise injurious germicidal agents. Preparatory preparation of the field of operation consists in scrubbing membranous, dental, and other surfaces, removal or antiseptic care of teeth or roots, and at least temporary stopping of carious tooth cavities. My post-operative sheet-anchor is dioxogen,[1] which gives mechanical cleansing in setting free the dead mucous cells and destroying the resistant nature of the intervening secretions, while at the same time it gives an immediate and powerful effect on bacteria in destroying their vital properties. Dioxogen and 2.5 per cent. carbolic acid should be used alternately once each hour during

[1] I use dioxogen because in my experience it has proved the most uniformly free from acid of any of the preparations of $H_2O_2$, commonly sold as such, and because an impure or a strong acid solution must necessarily be absolutely prohibited when hourly treatments of the mouths of patients, many of whom are infants, is prescribed.

the day, and at least four times at night. These washes are applied with a glass hospital syringe, with force enough to assist in the dislodgement of little particles of débris, but not force enough to be injurious.

In addition to hourly washing, the use of applicators, made in the usual way, of absorbent cotton wrapped on toothpicks, is required from three to four times daily to wipe surfaces that washing alone will not cleanse sufficiently. Almost from the moment of recovery from the anæsthetic, some one of the many oil preparations should be sprayed with a suitable nebulizer through the nasal passages, as well as the mouth, and continued throughout the treatment after each washing.

Fig. 48.

Copy of Scripture's photographs of sound-waves in "Who killed Cock Robin?"

In dealing with children and infants it is not always possible to follow all these directions, as they sometimes do not submit willingly, and naturally we must therefore do only so much as we safely can, but patience, judgment, and skill on the part of the nurse can do much to overcome such difficulties.[1]

[1] Other references which may be consulted are as follows: Le Dentia: La Médecine, June 5, 1890. Ehrmann: Les opérations plastiques sur le palais chez l'enfant, etc., Paris. Taylor: Dublin Journal of Medical Sciences, 1900. Edwin Owen: London Lancet, 1896. Telezit: Tribune Médicale, Paris, 1894. Talbot, Eugene S.: Stigmata of Degeneracy, and The Etiology of Osseous Deformities. Cryer, M. H.: Internal Anatomy of the Face. Fillebrown: Boston Journal of Medicine and Surgery.

## Figures and Tables

**Fig. 1. f1:**
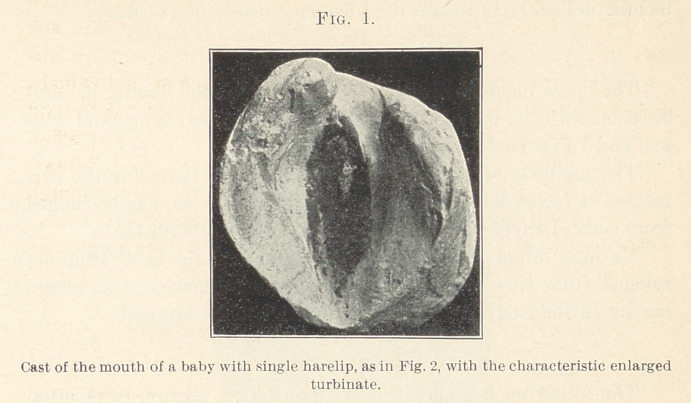


**Fig. 2. f2:**
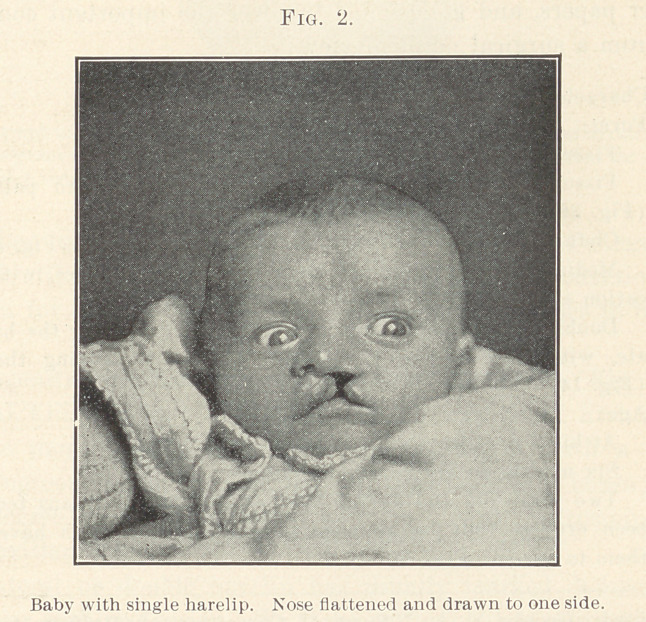


**Fig. 3. f3:**
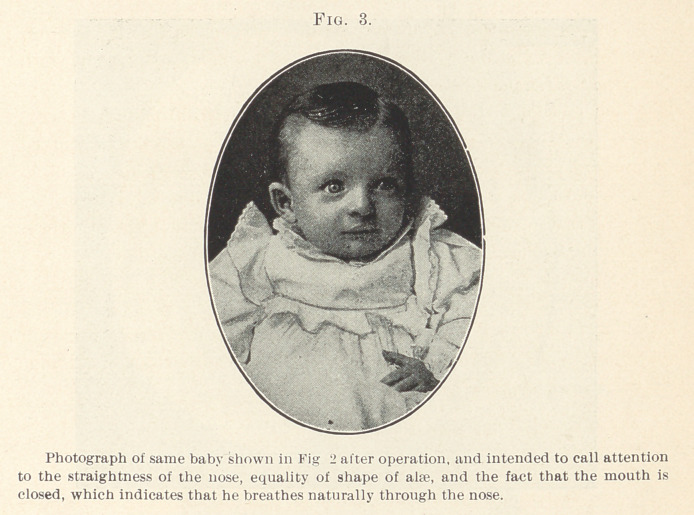


**Fig. 4. f4:**
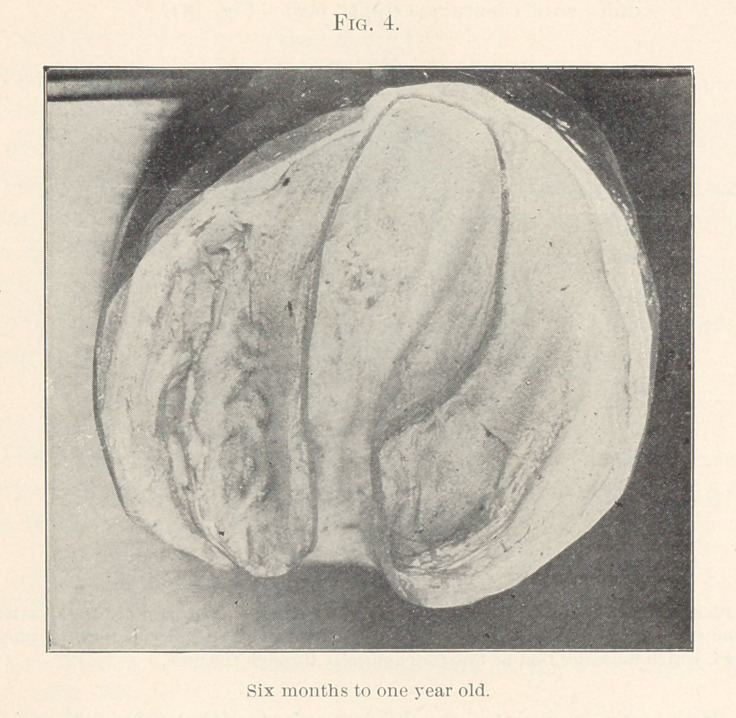


**Fig. 7. f5:**
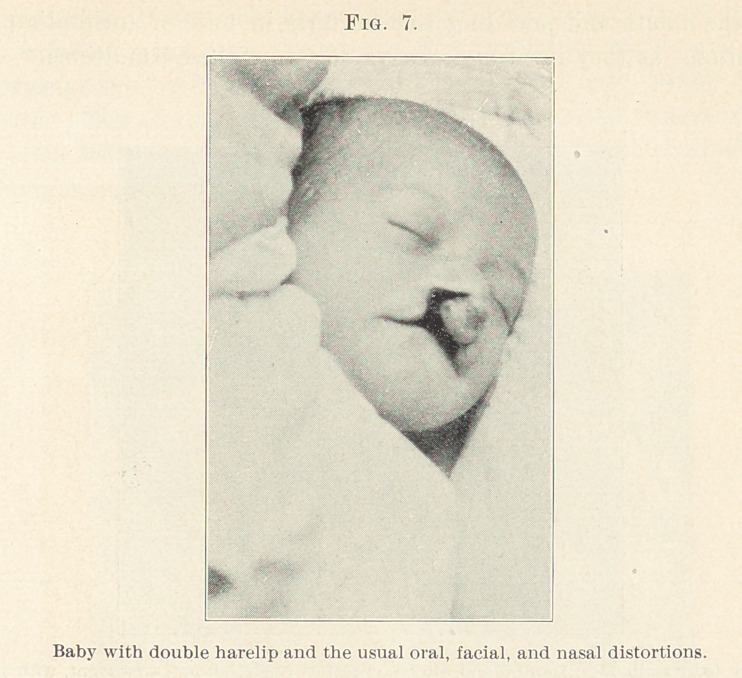


**Fig. 8. f6:**
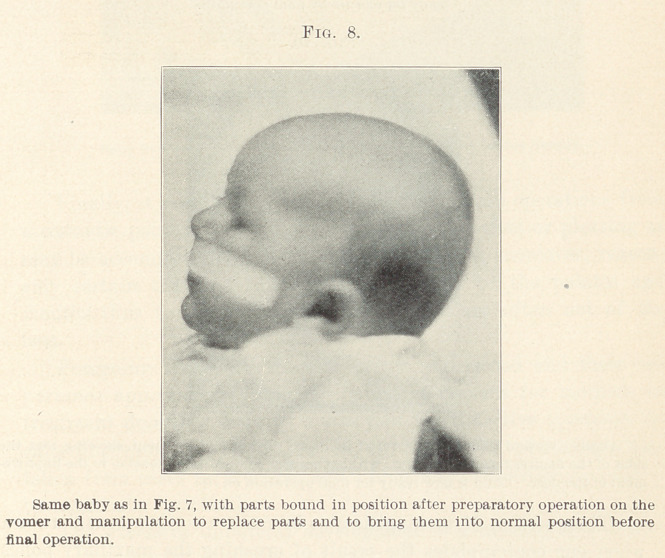


**Fig. 9. f7:**
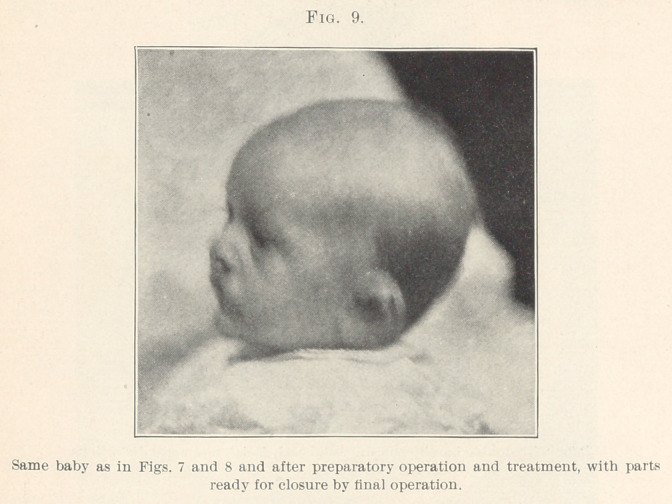


**Fig. 10. f8:**
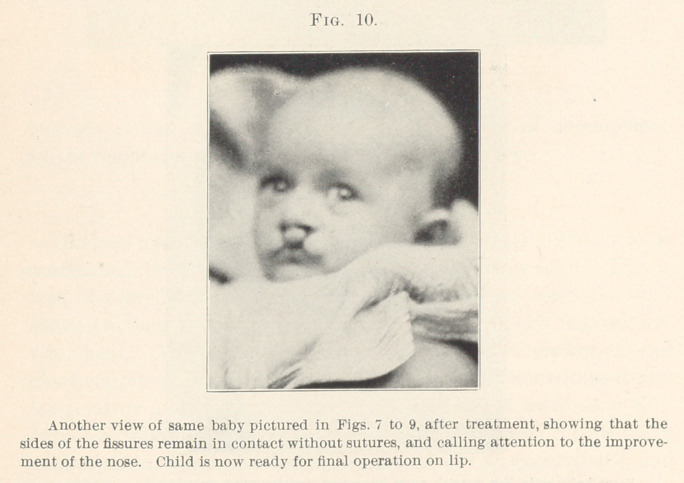


**Fig. 11. f9:**
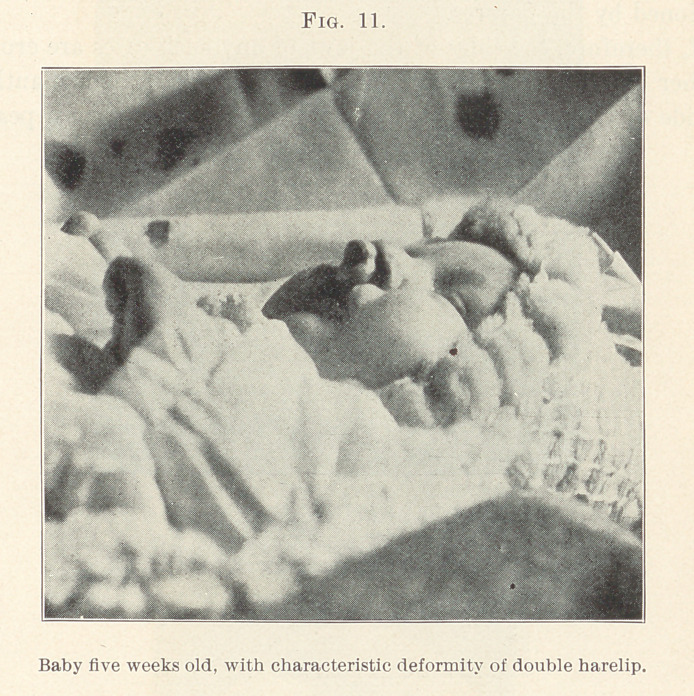


**Fig. 13. f10:**
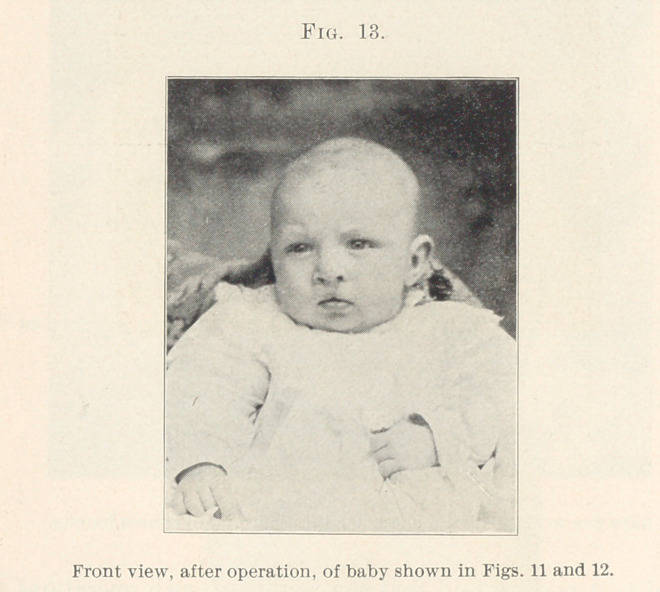


**Fig. 14. f11:**
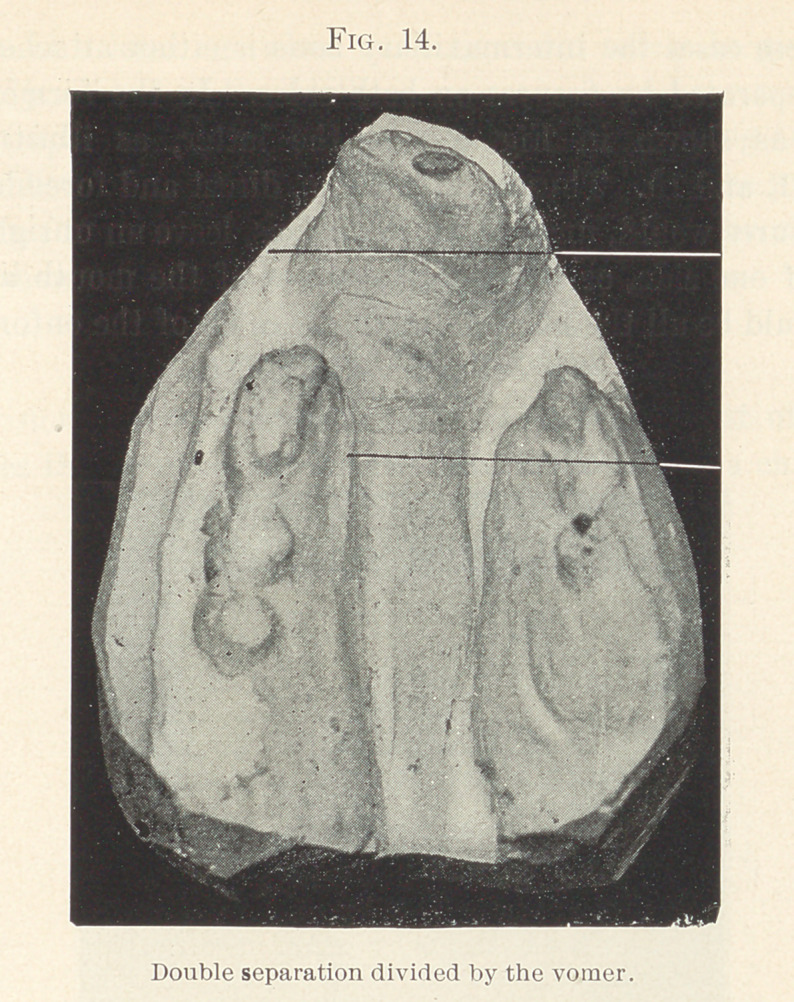


**Fig. 18. f12:**
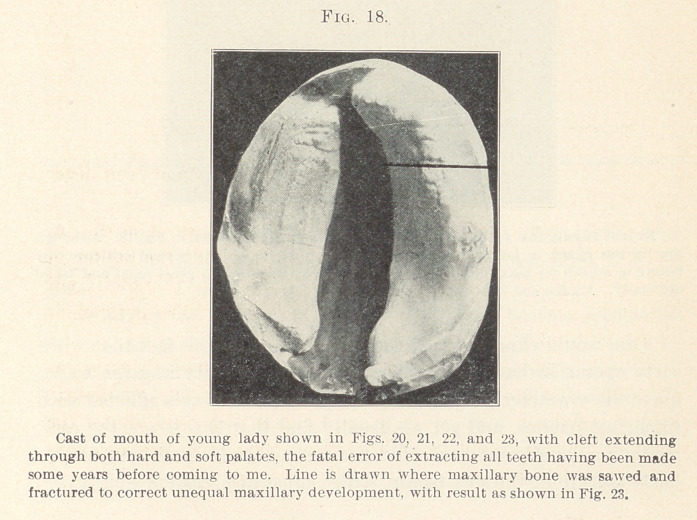


**Fig. 20. f13:**
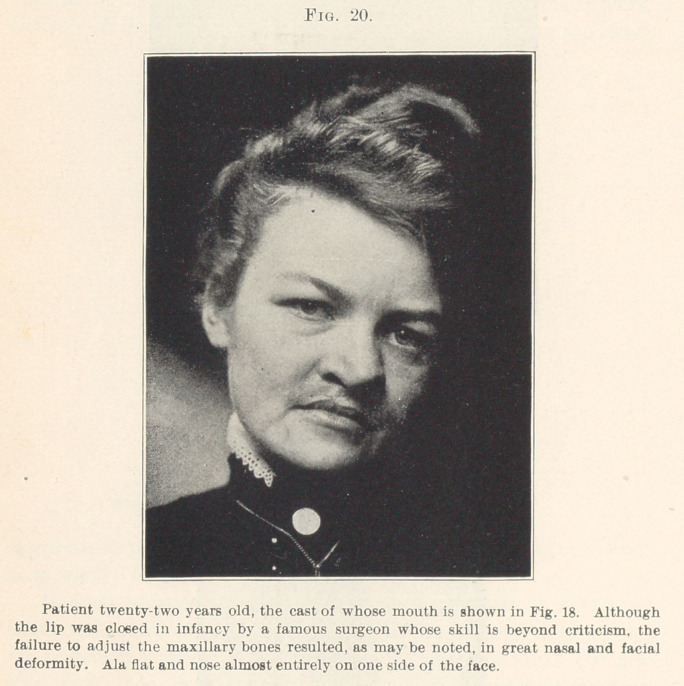


**Fig. 21. f14:**
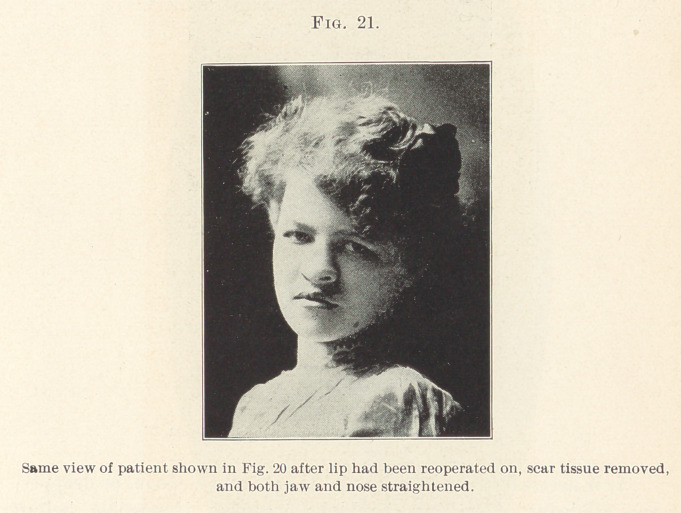


**Fig. 22. f15:**
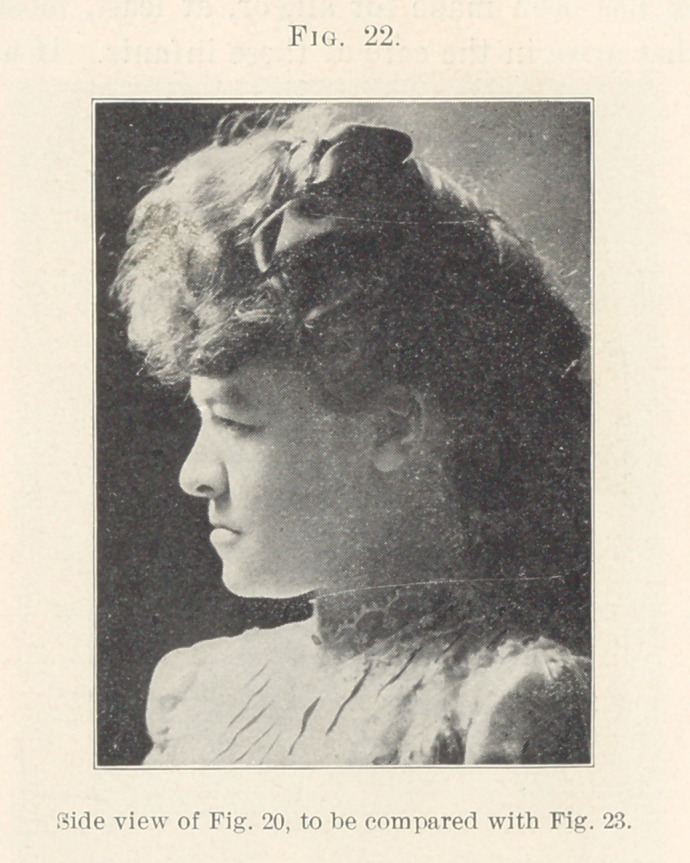


**Fig. 23. f16:**
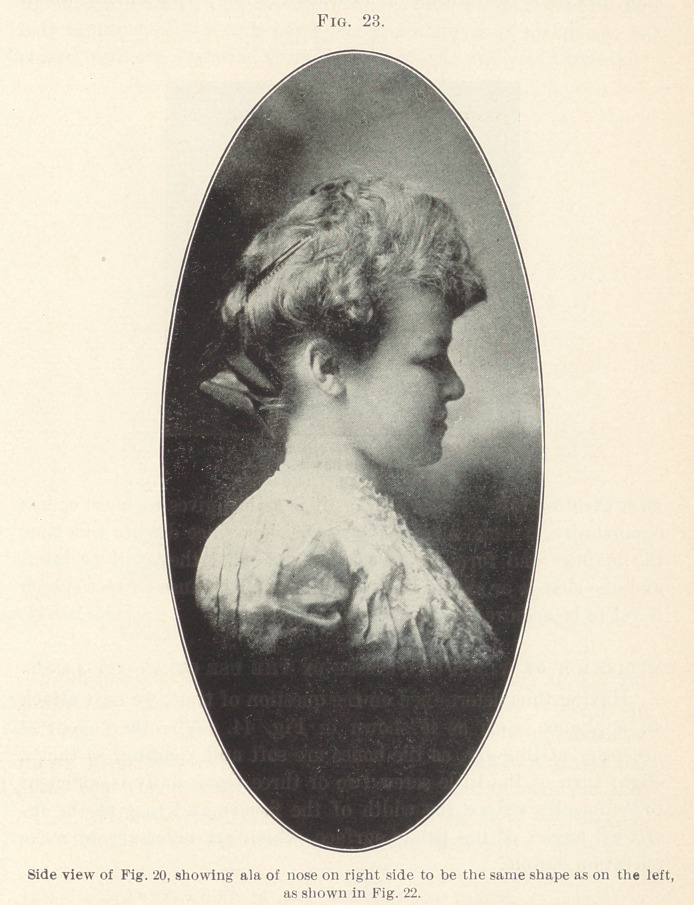


**Fig. 24. f17:**
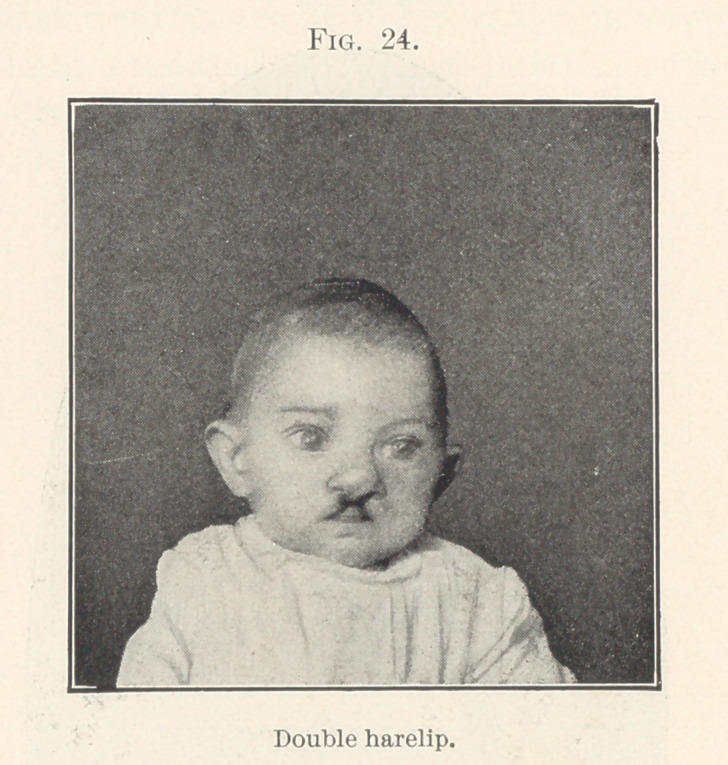


**Fig. 25. f18:**
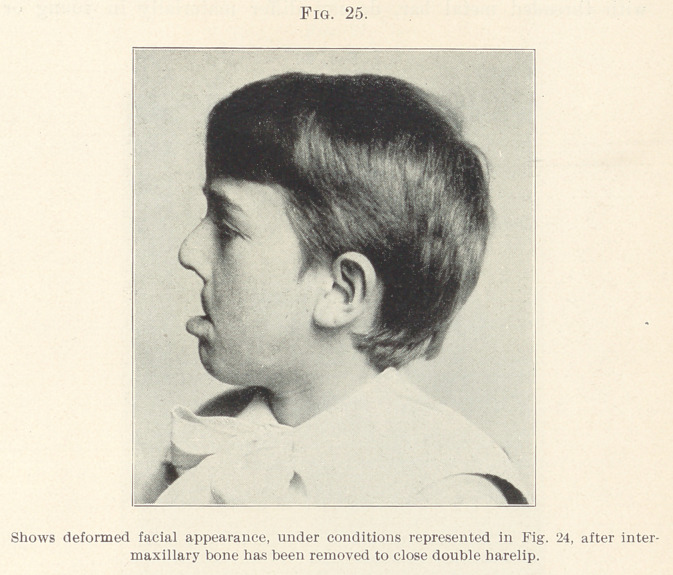


**Fig. 26. f19:**
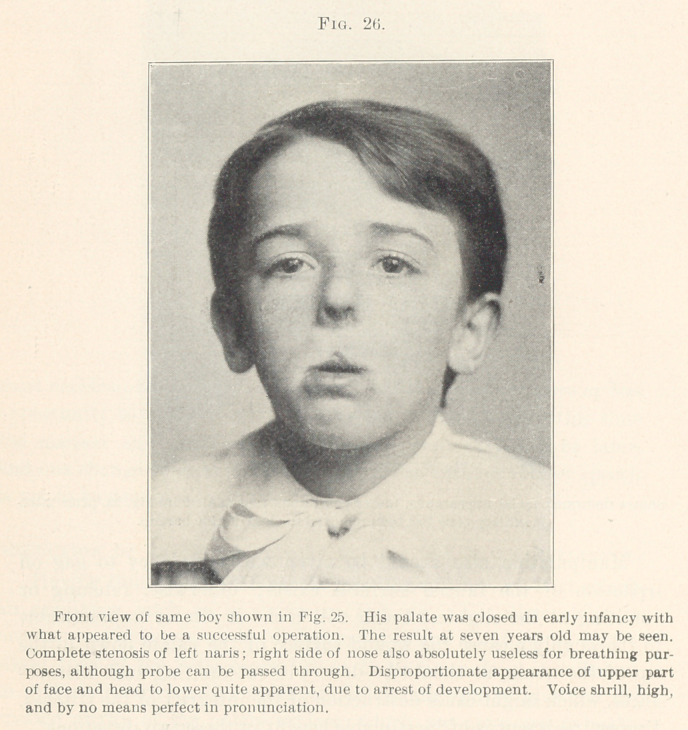


**Fig. 27. f20:**
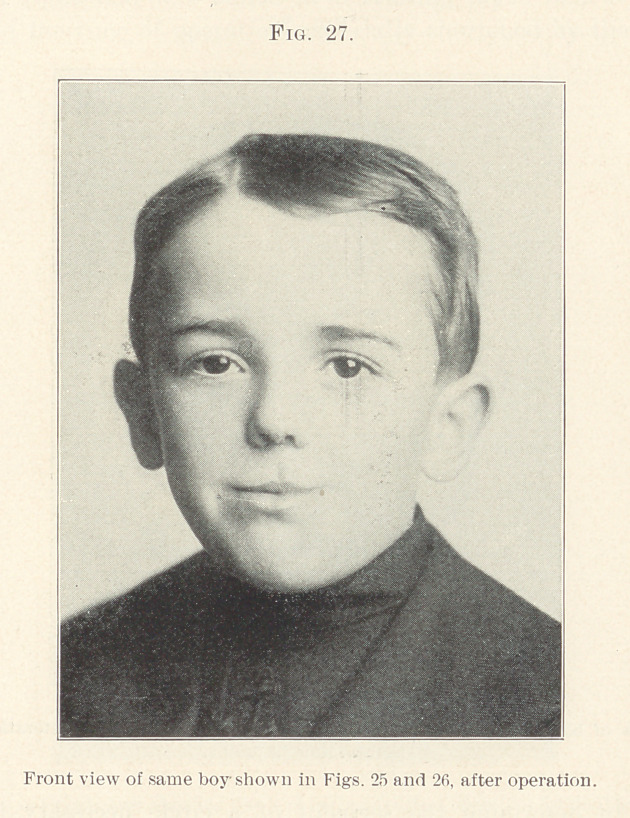


**Fig. 28. f21:**
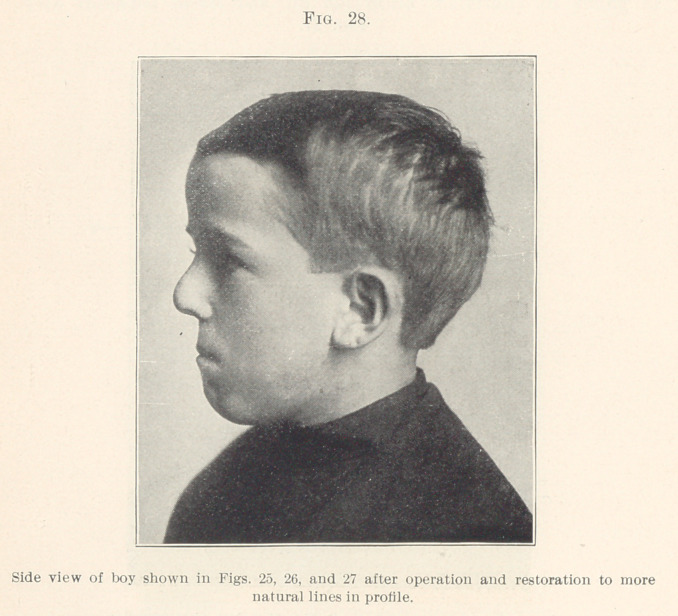


**Fig. 29. f22:**
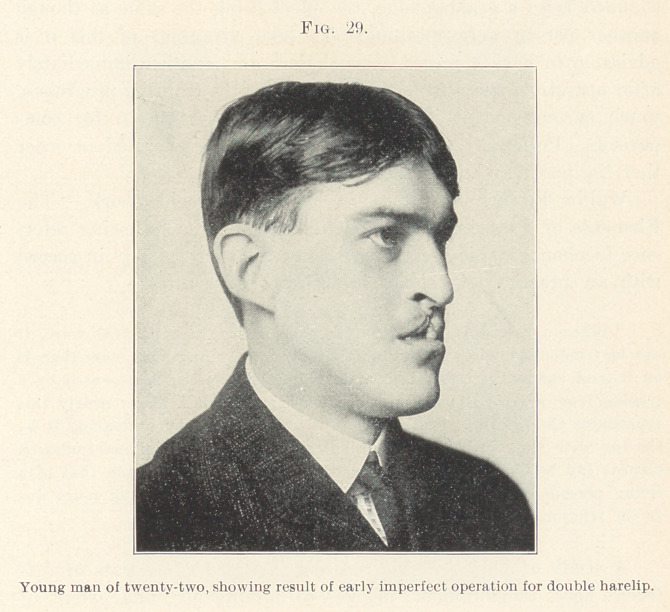


**Fig. 30. f23:**
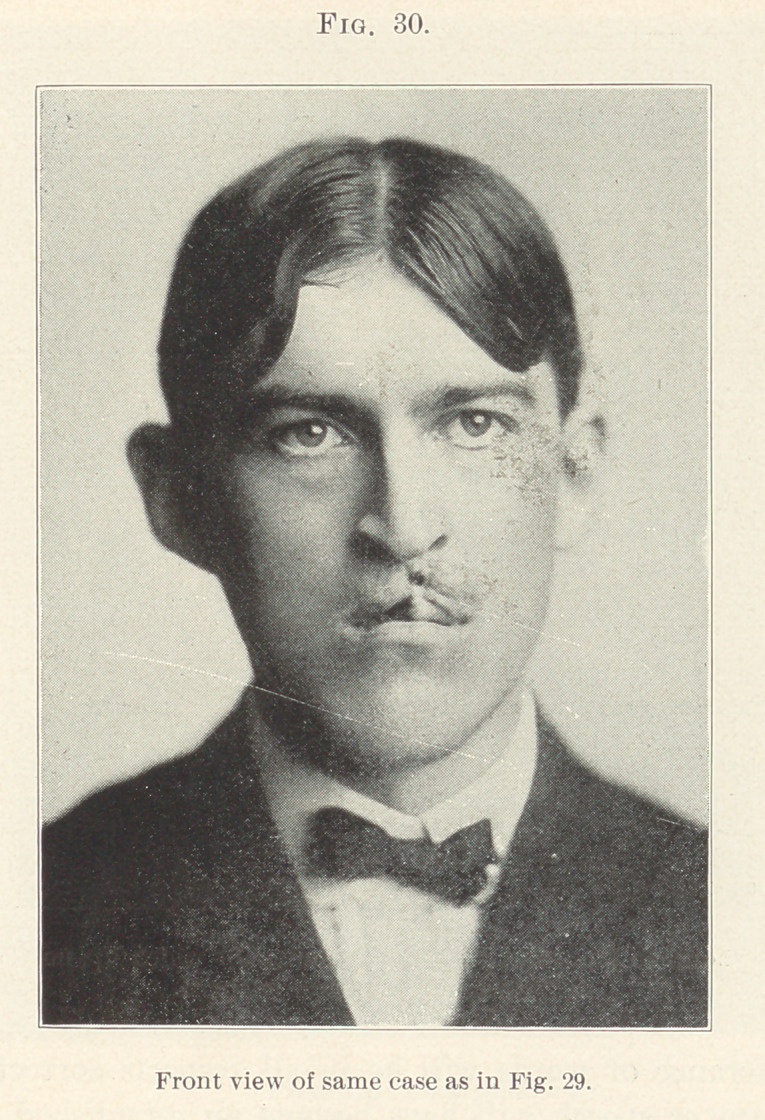


**Fig. 31. f24:**
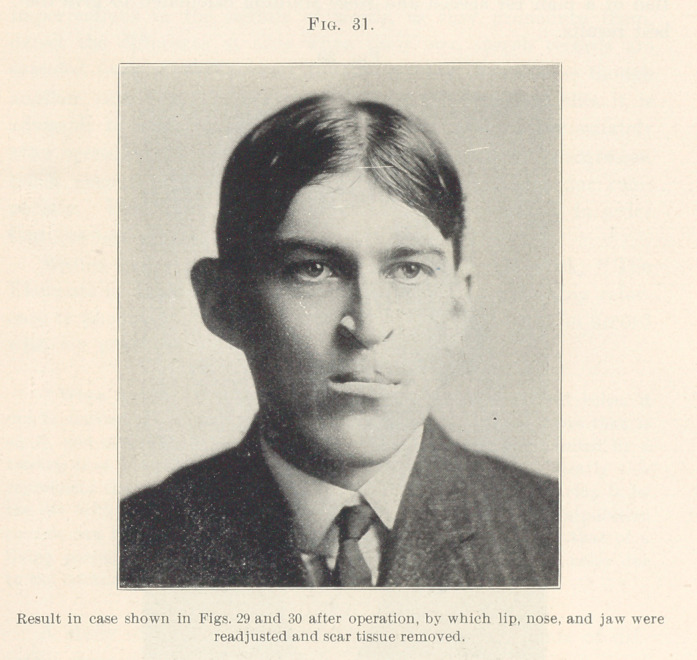


**Fig. 32. f25:**
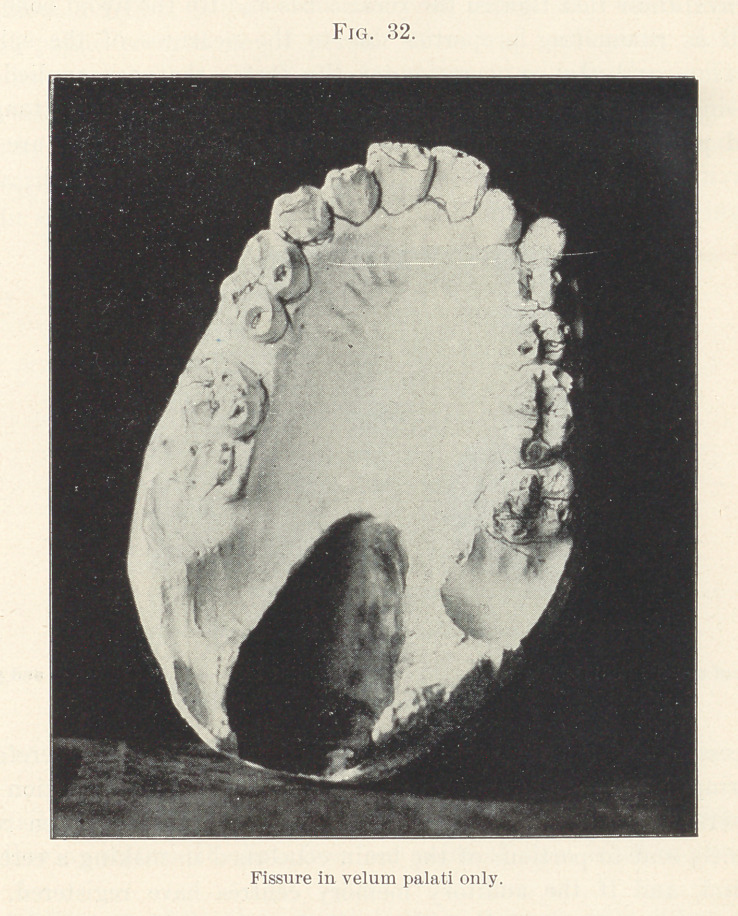


**Fig. 36. f26:**
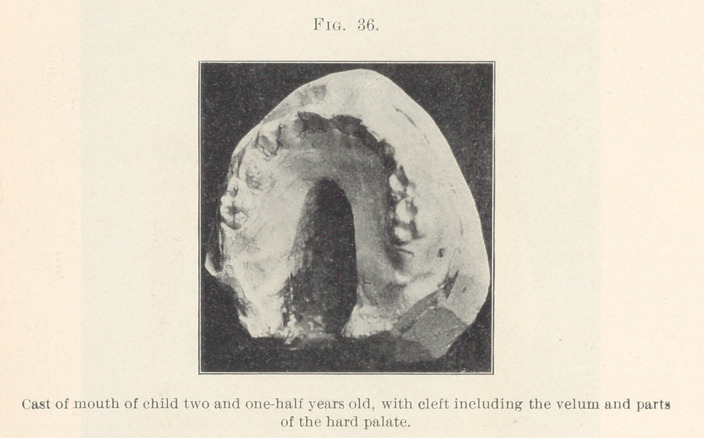


**Fig. 37. f27:**
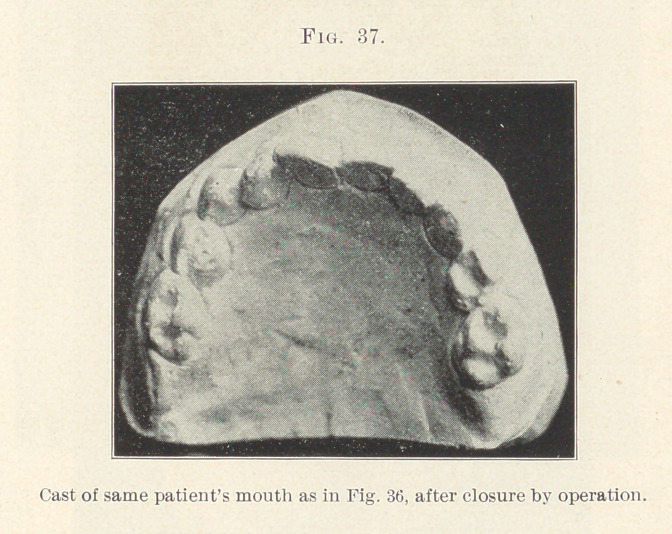


**Fig. 39. f28:**
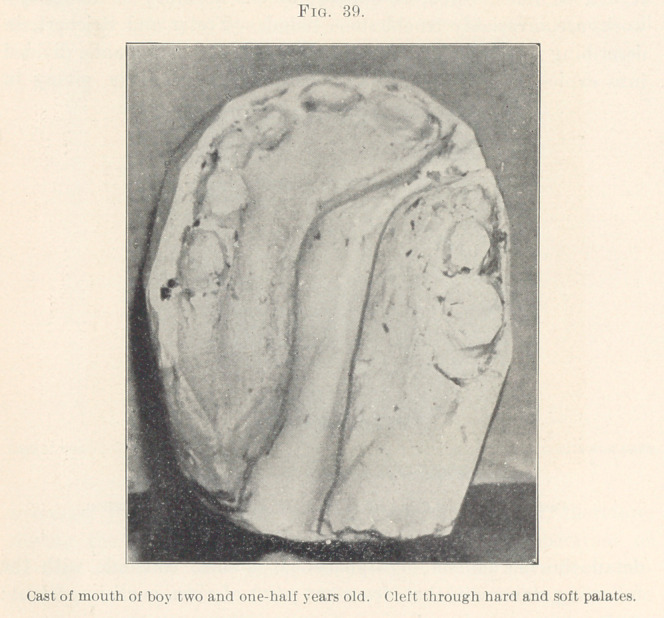


**Fig. 40. f29:**
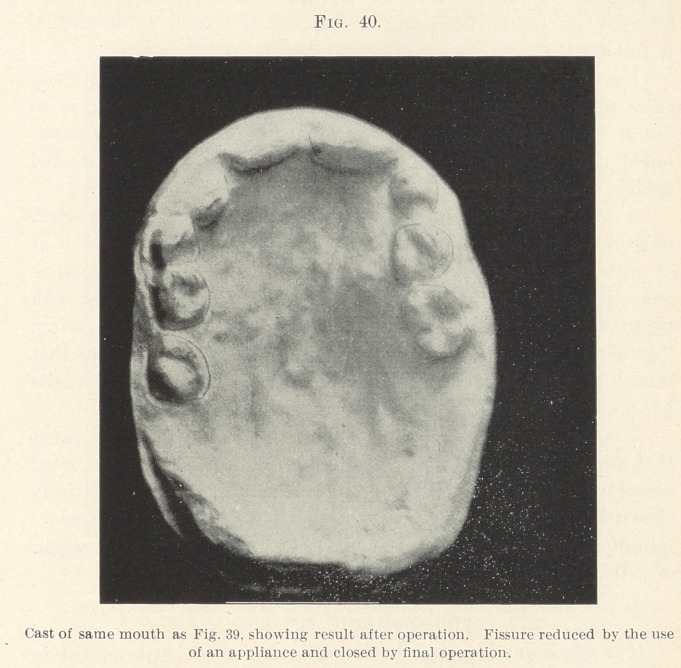


**Fig. 43. f30:**
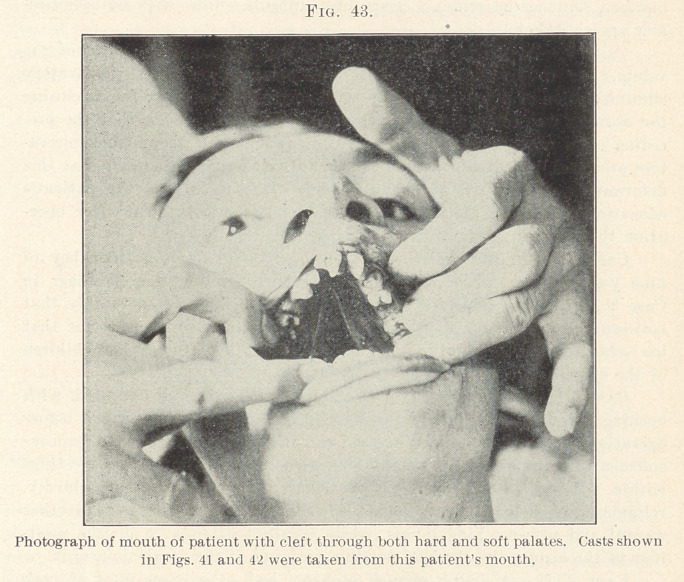


**Fig. 44. f31:**
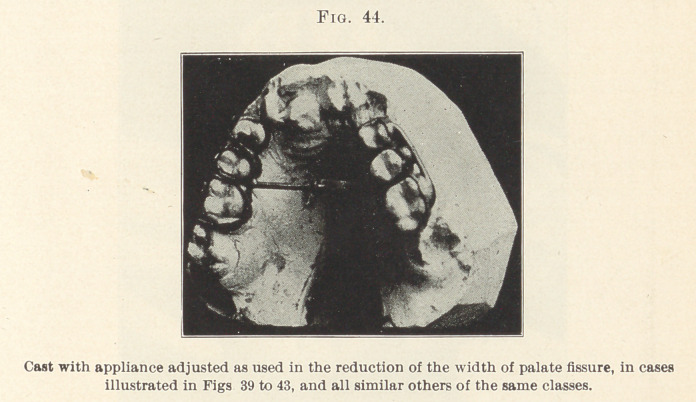


**Fig. 46. f32:**
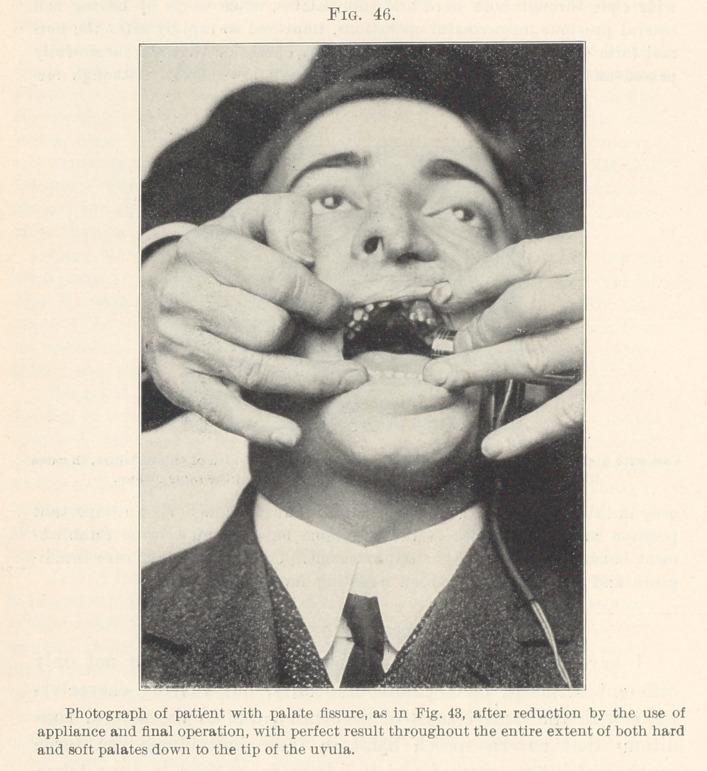


**Fig. 47. f33:**
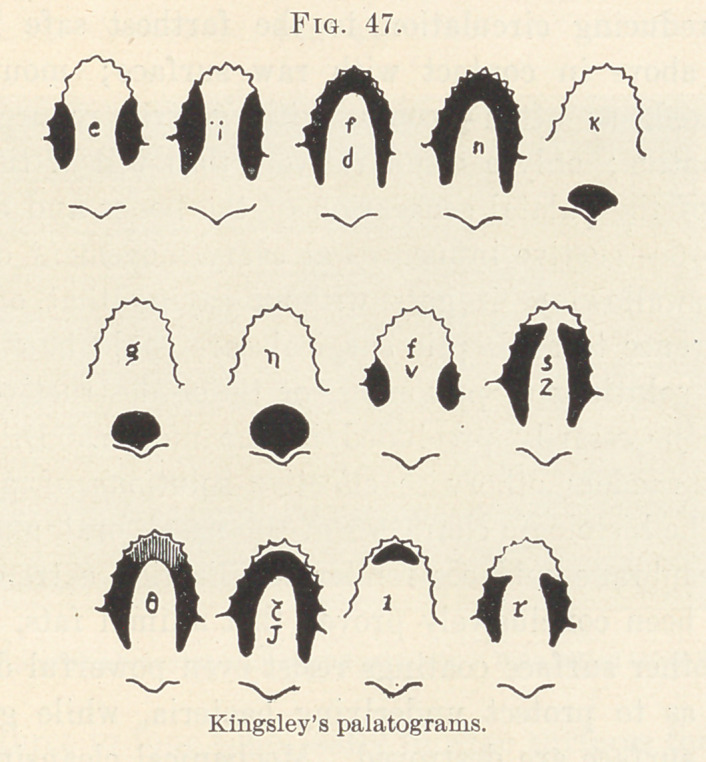


**Fig. 48. f34:**